# Dyeing Para-Aramid Textiles Pretreated with Soybean Oil and Nonthermal Plasma Using Cationic Dye

**DOI:** 10.3390/polym13091492

**Published:** 2021-05-06

**Authors:** Mary Morris, Xiaofei Philip Ye, Christopher J. Doona

**Affiliations:** 1 Department of Biosystems Engineering and Soil Science, The University of Tennessee, Knoxville, TN 37996, USA; maramorr@vols.utk.edu; 2 U.S. Army Combat Capabilities Development Command—Soldier Center, Natick, MA 01760, USA; doonac@mit.edu; 3 Research Affiliate, Massachusetts Institute of Technology—Institute for Soldier Nanotechnologies, 77 Massachusetts Ave., NE47-4F, Cambridge, MA 02139, USA

**Keywords:** soybean oil, glycerol, nonthermal plasma, para-aramid textiles, cationic dye

## Abstract

The increasing use of functional aramids in a wide array of applications and the inert nature of aramids against conventional dye and print methods requires developing new dyeing methods. This study aims to use environmentally friendly method with a cationic dye as an alternative for dyeing para-aramid fabrics. Experiments used a multi-factorial design with functions of pretreatment, dye solvent (water and/or glycerol) and auxiliary chemical additives (swelling agent and surfactant) and a sequential experimentation methodology. The most effective dyeing procedures involved the following steps: (i) pretreatments of the fabrics with soybean oil and nonthermal plasma (NTP), (ii) using water at T = 100 °C as the dye solvent, and (iii) omitting other chemical additives. With a commercial cationic dye, these conditions achieved a color strength in K/S value of 2.28, compared to ~1 for untreated samples. FTIR analysis revealed that a functional network formed on the fibers and yarns of the fabrics by chemical reactions of excited plasma species with double bonds in the soybean oil molecules was responsible for significantly improving the color strength. These results extend the potential uses of a renewable material (soybean oil) and an environmentally friendly technology (NTP) to improve the dyeing of para-aramid textiles and reduce the use of harsh dye chemicals.

## 1. Introduction

Aramid (portmanteau combining aromatic and polyamide) fibers are high-performance fibers with properties of a high tensile strength, high melting point, chemical resistance to a range of organic solvents and exceptional flame resistance [1]. Consequently, aramid fibers have found wide applications in various technical areas, including textiles for apparel such as body armor (bullet-proof vests and helmets), puncture–resistant correctional wear, fire-protective clothing and sportswear and other applications such as brake pads, gaskets, hot-air filters, industrial belts and ropes, reinforced composites, tire cords and the strength member in fiber optics.

There are two main types of aramids: meta-aramids and para-aramids, and these two groups have different properties due to differences in their molecular structures. At the fundamental level, para-aramid fibers consist of poly(p-phenylene terephthalamide) molecules estimated to be 230 nm long, with stiff para-linked aromatic rings and densely arranged hydrogen bond donors and acceptors throughout their backbones. This inherent molecular rigidity, combined with strong intermolecular hydrogen bonding interactions, enables the molecules to achieve excellent alignment with their neighbors, resulting in a highly anisotropic unit cell consisting of covalent bonds, hydrogen bonds and van der Waals interactions along each fundamental axis, forming a highly crystalline structure [1]. The building-block molecules of meta-aramid are poly(m-phenylene isophthalamide) that bind via meta-linked aromatic rings to result in a semi-crystalline fiber with the molecular chain oriented along the fiber axis.

With the increasing array of applications using aramids, there is also the need to find alternative methods for dyeing aramid textiles. It has been a significant challenge to dye or print aramid fabrics to a high color strength using conventional dyeing and printing methods, especially for continuous filament para-aramids due to their highly crystalline structure and chemical inertness. Currently, the color of woven aramid materials uses primarily solution dyeing methods, in which the coloring of the yarns in the woven or knit fabric is determined by adding colorant to the polymer dope at the time the aramid filament is produced, thereby limiting the color options for the fabrics and their use in potential new applications. Various surface modification methods have been attempted to improve the dyeing of aramids, including chemical treatments with strong acids [2,3] and auxiliary additives [4], physical approaches using UV/O_3_ irradiation [5] or nonthermal plasma [6], and chemical grafting using poly(acrylic acid) [7] or a diblock copolymer derived from methacryloyloxy-ethyl-trimethylammonium chloride [8], which claimed improved dyeability with different types of dyes. Most of these methods focused on the surface modification of meta-aramids, and few reported successes in dyeing para-aramids [9,10,11].

In terms of the dye characteristics suited for dyeing aramids, disperse dyes were developed to dye synthetic fibers and worked well on nylon [12], which were aliphatic or semi-aromatic polyamides, but limited success had been reported in dyeing aramids without harsh chemical treatments [4]. In solution dyeing, the pigments are added to the sulfuric acid solution just before spinning to achieve coloration of the resultant para-aramid fibers [13]. Further, disperse dyes are so-called because they tend to have low solubility in water and require special dispersing agents to effectuate coloration. Another type of dye, the cationic dyes, are exclusively used in solution dyeing, with the help of special dyeing auxiliaries and/or solvents in the case of meta-aramid fibers. In contrast, cationic dyes can dissociate into positively charged chromophore ions in aqueous solution and interact with the negative groups on the fiber molecules to form salts, which can become firmly attached to the fibers for dyeing. Using conventional dyeing methods to investigate dyeing characteristics of meta-aramid fibers with some commercial dyes, Kim and Choi [14] reported that the cationic dyes showed comparatively higher exhaustion yield comparing to those of disperse dyes and acid dyes, and under acidic conditions in the range of pH 3 to 5, the stability of cationic dyes could be enhanced, leading to higher adsorption.

We hypothesize that surface modification of para-aramids via nonthermal plasma (NTP) treatment, particularly an oxygen-containing plasma method, would introduce anionic dyeing sites on the surface to facilitate dyeing. Therefore, the objective of this study is to dye para-aramid textiles with a cationic dye using renewable materials and an environmentally friendly technology (NTP). Additionally, since the trend in the textile dyeing industry is to reduce or avoid the use and disposal of environmentally-unfriendly chemical additives [15], glycerol is used in this study as an alternative solvent to water or ethanol due to glycerol’s non-toxicity, low volatility and high boiling point (290 °C), all of which make glycerol environmentally safe and suitable for use in the textile dyeing industry [16,17]. Further, glycerol is also used in this study, because there is a current worldwide effort to valorize the excessive amount of crude glycerol that has been generated as a result of massive biodiesel production [18,19,20,21].

Textile dyeing processes commonly add surfactants to the dyeing media, to ensure the uniform dispersion of dye in the media that promotes penetration of the dye into the fiber matrix [22]. Swelling agents or dyeing accelerators swell the fibers to facilitate the penetration of dye into the fibers. Swelling agents are particularly useful for increasing the dye substantivity in some instances with highly crystalline synthetic and blended fibers [23,24]. In this study, the nonionic surfactant and emulsifier Polysorbate 80 (TWEEN 80) and the swelling agent benzyl alcohol, both of which have been reported to have benefits in dyeing aramids [25,26], were tested as additives to improve dyeing.

## 2. Materials and Methods

### 2.1. Materials

The textiles tested in these experiments consisted of continuous filament 300 Denier para-aramid yarns in a tightly woven plain weave construction, as provided by the U.S. Army Combat Capabilities Development Command—Soldier Center (Natick, MA, USA). The cationic dye Basic Blue 11 (Victoria Blue R, CAS Number 2185-86-6) was purchased from Sigma-Aldrich (St. Louis, MO, USA); glycerol (99.5% purity) and acetic acid (glacial) were purchased from Fisher Scientific (Waltham, MA, USA); Polysorbate 80 (TWEEN 80) and benzyl alcohol were purchased from Chem Center @ Amazon.com; and food grade refined soybean oil (100 g contains approximately 16 g of saturated fat, 23 g of monounsaturated fat, and 58 g of polyunsaturated fat) and laundry detergent (ECOS^®^ Plus with Stain-Fighting Enzymes) were purchased from a local supermarket. All reagents and materials were used as-is without further purification.

### 2.2. Experimental Methods

The sequential experimentation methodology used in this study (Experiments A–F) was directed toward the goal of finding the combination of experimental factors that achieve the highest color strength of para-aramid fabrics dyed with a cationic dye. The experimental design used sequential factorial designs to quickly identify critical factors and conditions that improved dyeing. The factorial functions consisted of: (i) pretreatment with acetic acid, soybean oil and/or NTP treatment, (ii) dye solvent using water versus glycerol and (iii) the addition of the auxiliary additives of swelling agent and surfactant. The experimental design and subsequent analysis were carried out using specially designed software Design-Expert 6.0 (Stat-Ease, Inc., Minneapolis, MN, USA).

### 2.3. Pretreatment (Acetic Acid, Soybean Oil, NTP)

Para-aramid textile fabric samples (~645 mm^2^ or ~1 in^2^) were pretreated by simply immersing the fabric samples into a Petri dish containing either 20 wt.% aqueous acetic acid solution or soybean oil, according to an experimental design. The samples were removed after 15 h, transferred onto a paper towel and hand-pressed with a roller to force out any excess liquid.

For fabric samples subjected to NTP exposure, the NTP treatment was carried out using an in-house made Surface Dielectric Barrier Discharge (SDBD) system. The SDBD method was chosen because it generates a higher density of micro-discharges that are limited to the sample surface compared to the Volume Dielectric Barrier Discharge (VDBD) method. The SDBD therefore avoids the formation of pinholes in the para-aramid fabric samples, which is commonly associated with VDBD and caused by hot electron bombardment of the para-aramid fabrics. Teflon-coated aramids have been made wettable after a 30 s exposure to this SDBD.

**Figure 1:**
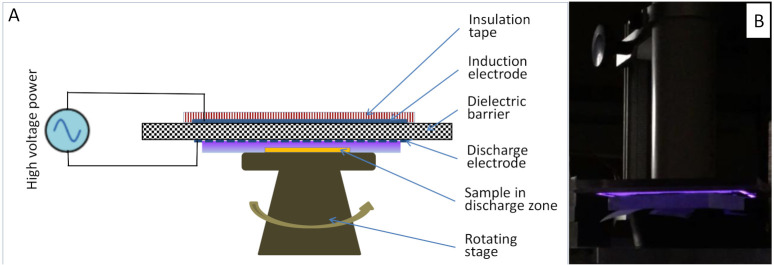
(**A**) Diagram of the NTP system and (**B**) photo showing a sample under glowing plasma discharge.

The SDBD system has been described previously for dyeing with a disperse dye [27] and is summarized here. The SDBD apparatus uses ambient air as the feed gas and comprises two electrodes that are separated by an alumina dielectric plate (dimensions = 108 × 95 × 1 mm^3^ thick) and powered by a high voltage power source with a sinusoidal high voltage of 9.2 kV that was tuned to a 23.2 kHz resonance frequency (see Figure 1). Embedded at the top of the alumina plate was an induction electrode made of rectangular copper tape, and at the bottom of the alumina plate was a discharge electrode (17 tungsten strips interconnected). Fabric samples were placed on the rotating stage and the SDBD plate was lowered to a height 1 mm above the sample. When the power was switched on, the plasma emissions, which included reactive oxygen and nitrogen species (RONS) and other high-energy radicals, interacted with the fabric samples (typically for 1–2.5 min). Then, the power was turned off and the sample was removed for dyeing.

### 2.4. Dyeing Experimental Procedures

The cationic dye Victoria Blue R is soluble in water and in glycerol, so distilled water, glycerol and aqueous glycerol solutions were tested and compared as the dye solvent. The aqueous glycerol solutions are expressed as their volumetric percent of glycerol. The dye solvents were moderately heated (T ≈ 50 °C) with vigorous stirring using a magnetic stirrer, and appropriate amounts of Victoria Blue R dye, acetic acid, surfactant and swelling agent were added to the respective solvents in accordance with the design of the experiment. The dye bath liquid was dispensed into separate vials that were simultaneously heated and stirred on a Reacti-Therm system (ThermoFisher Scientific, Waltham, MA, USA). Fabric samples were loaded into the vials for dyeing. The dye bath and fabric samples took about 30 min to reach a designated temperature, held at the temperature for 1 h, then cooled for 20 min with the Reacti-Therm system shut off. After the dyeing, samples were rinsed in flowing warm tap water for 2 min, rinsed in cold water for 2 min, then dried in a programmable convective oven at T = 150 °C for 2 min (after starting at T = 30 °C and increasing the temperature at a rate of 30 °C/min) to fix the dye.

### 2.5. Color Strength Analysis

Prior to determining the color strength, the fabric samples were washed with laundry detergent using a homemade tumbler, in accordance with the ISO standard 105-C10:2006 protocol [28], then rinsed and dried as described in Section 2.4 above.

Estimates of the color strength for each of the dyed fabric samples were determined at the wavelength of maximum absorbance for Victoria Blue R dye (λ_max_ = 615 nm) by measuring percent spectral reflectance (%R) in the visible range with a SPECTRO 1 spectrophotometer (Variable Inc., Chattanooga, TN, USA). Results were expressed as the samples’ absorption (K) and scattering (S) characteristics as its K/S value, which varies approximately linearly with colorant concentration, according to the Kubelka-Monk equation (Equation (1)) [29].

(1) $$K/S=\frac{{\left(1-0.01R\right)}^{2}}{2\left(0.01R\right)}$$

### 2.6. FTIR Analysis

In order to monitor some of the chemical changes occurring during the pretreatment and dyeing processes, Attenuated Total Reflection—Fourier Transform Infrared (ATR-FTIR) spectroscopy was used to analyze the fabric samples at different stages of the pretreatment and dyeing processes with the Victoria Blue R dye. In summary, fabric samples were placed on a potassium bromide (KBr) plate, pressed under the germanium crystal of ATR (UMA 400, Varian Inc., Palo Alto, CA, USA) and scanned in the mid-IR region (500–4000 cm^−1^ with a 4 cm^−1^ resolution) with an FTIR spectrometer (Excalibur 3100, Varian Inc., Palo Alto, CA, USA) equipped with an Attenuated Total Reflection (ATR) accessory. The ATR-FTIR spectra were displayed in absorbance units with each spectrum representing an average of 128 scans and taking into account the background spectrum acquired using a blank KBr plate.

## 3. Results and Discussion

### 3.1. Experiments A–F: Analysis

Experiment A was a 4-factor, 2-level, full factorial experiment designed to probe the effects of dye solvent (water or glycerol), pretreatment with acetic acid and/or NTP treatment for 60 sec and the addition of the swelling agent benzyl alcohol. The dyeing results are also shown in Table 1.

**Figure 2:**
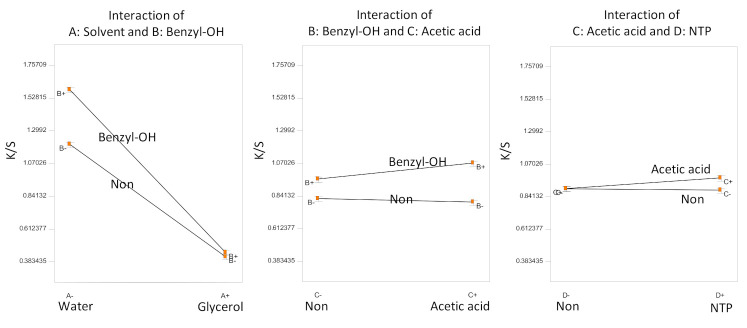
Statistical inference of Experiment A: significant factorial interactions.

Overall, the highest K/S value achieved in Experiment A is only 1.74 (sample A7) through the combination of water as solvent, pretreatments with acetic acid and NTP (60 s) and the addition of swelling agent benzyl alcohol. The Analysis of Variance (ANOVA) factorial model terms were selected based on the half-normal probability plot, which indicated that all the factors and some of their interactions were significant (*t*-tests for coefficients, *p* < 0.02). Since all of the factors are involved in interactions, statistical interpretations of only the significant interaction terms are shown in Figure 2.

Evidently in Figure 2, the dye solvent had the most significant influence on the color strength (K/S value), with water performing significantly better than glycerol. Adding benzyl alcohol to the dye bath increased the color strength, and the effect of benzyl alcohol was slightly enhanced by the acetic acid pretreatment with water as the dye solvent. The effects of pretreatment with acetic acid and NTP were not obvious, slightly improving the color strength with their synergy.

A significant loss of water was observed in the dye bath during Experiment A due to the evaporation of water at the dyeing temperature T = 140 °C. The use of glycerol as the dye solvent effectuated dyeing at T = 140 °C without the concomitant evaporative loss of solvent from the dye bath but reached significantly lower values of color strengths. The cationic dye might not be well-dissociated in glycerol as the solvent, and the high viscosity of pure glycerol compared to water and the occurrence of hydrogen bonding between the dye and glycerol might hinder the mobility of the cationic dye and retard its diffusion onto the para-aramids.

To overcome the drawbacks of using glycerol as the dye solvent, full factorial Experiment B was designed using 50% or 80% aqueous glycerol solutions as the dye solvent. Additional factors were investigated and included a pretreatment of soaking the para-aramid fabrics in soybean oil and/or applying further treatment with NTP and adding benzyl alcohol as the swelling agent. Table 2 lists the detailed experimental design and resultant K/S values. Even though using 50% or 80% aqueous glycerol solutions as the dye solvent suppressed the loss of the dye bath solution, the highest K/S value achieved in this experiment was only 0.94 (sample B11) with 50% aqueous glycerol solution as the solvent and soybean oil and NTP (60 s) as the pretreatment.

**Figure 3:**
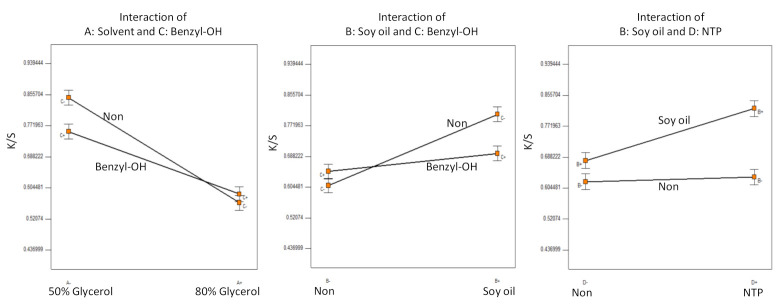
Statistical inference of Experiment B: significant factorial interactions.

The analysis of variance (ANOVA) of this factorial design indicates that the main effect of all the factors and some of their interactions are significant (*p* < 0.005). The half-normal probability plot was used to select the ANOVA model terms, and only the significant interaction terms of the model are presented in Figure 3.

The 50% aqueous glycerol solution performed better than the 80% aqueous glycerol solution as the dye solvent (Figure 3). Benzyl alcohol appeared to be incompatible with aqueous glycerol or soybean oil, which might hinder its diffusion and swelling functions and consequently limited the benefits of soybean oil on dyeing. Soaking the para-aramids in soybean oil slightly improved the dyeing color strength, while soaking the para-aramids in soybean oil with subsequent NTP treatment for 60 s increased the color strength significantly. Without a pretreatment of soybean oil pretreatment, NTP had negligible effect on the dyeing strength.

The full factorial design for Experiment C used a lower dyeing temperature (T = 90 °C), compared 50% aqueous glycerol solution with water as the dye solvent and used soybean oil pretreatments and NTP treatment times of 90 s. The experimental design and dyeing results are compiled in Table 3. The highest K/S value achieved in this experiment is 1.72 (sample C11), which is higher than the highest value obtained in Experiment B (0.94). Experiment B was carried out at a higher temperature (T = 140 °C vs. T = 90 °C for Experiment C), used soybean oil with a shorter NTP treatment time (60 s vs. 90 s for Experiment C) and used 50% or 80% aqueous glycerol solutions as the dye solvent (vs. water or 50% aqueous glycerol in Experiment C).

**Figure 4:**
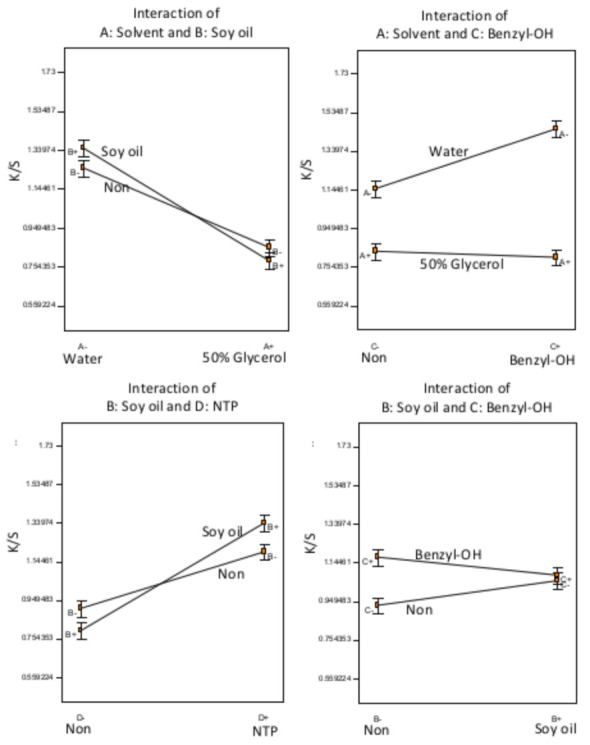
Statistical inference of Experiment C: significant factorial interactions.

The half-normal probability plot was used to select the ANOVA model terms and resulted in a significant model (F-test, *p* < 0.0001). All of the terms included in the model were significant (*t*-tests, *p* < 0.007), except for the main effect of swelling agent (*t*-test, *p* = 0.59). Since all of the factors were involved in interactions, only the significant interaction terms are interpreted in Figure 4.

According to the half-normal probability plot, the solvent had the most influence on color strength among all of the factors, with water as the solvent performing significantly better than the 50% aqueous glycerol solution. Soybean oil pretreatment followed by NTP significantly improved the color strength, although NTP treatment without the soybean oil pretreatment also improved the color strength, but to a lesser degree. Benzyl alcohol performed better in water than in 50% aqueous glycerol solution as the solvent, another indication that the benzyl alcohol was not compatible with glycerol in Experiment B. Further, the effect of benzyl alcohol was minimal on the samples pretreated with soybean oil, but benzyl alcohol significantly improved dyeing without the soybean oil pretreatment, also indicating that benzyl alcohol was not compatible with soybean oil. Without a subsequent NTP treatment, soybean oil soaking resulted in slightly lower color strength, which seems contradictory to the results in Experiment B. However, Experiment C was carried out at a much lower temperature (T = 90 °C vs. T = 140 °C in experiment B with water or 50% aqueous glycerol solution (vs. 50% or 80% aqueous glycerol in Experiment B in the dye bath, and these factors may have limited the beneficial effects of the soybean oil in achieving dyeing without an NTP treatment. The longer NTP treatment time in Experiment C (90 s vs. 60 s in Experiment B also significantly improved dyeing even without the soaking in soybean oil pretreatment.

From the results of the previous Experiments A–C, it is clear that the use of glycerol in the dye solvent does not help with dyeing, and there is a potential for improving dyeing using a pretreatment consisting of soaking in soybean oil followed by NTP. Accordingly, Experiment D used a lower dyeing temperature (T = 90 °C), water as the dye solvent, a wider range of NTP treatment times (*t* = 30–150 s), eliminated the swelling agent benzyl alcohol as a factor and added surfactant as a new factor using TWEEN 80, because of its reported benefit in dyeing aramids [25,26]. Results of the 3-factor 2-level full factorial design are presented in Table 4.

**Figure 5:**
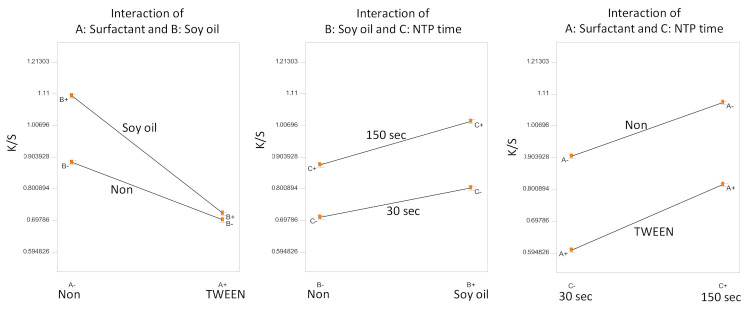
Statistical inference of Experiment D: significant factorial interactions.

ANOVA for the factorial design indicated a significant model (*F*-test, *p* < 0.0001). All three of the factors and their two-way interactions were significant (*t*-test, *p* < 0.0002). Since all of the factors are involved in interactions, only the interaction plots are shown in Figure 5.

According to the half-normal probability plot, the surfactant TWEEN 80 had the highest impact on the dyeing color strength, followed by NTP treatment time, then soybean oil pretreatment in descending order. The use of TWEEN 80 resulted in weaker color strength (Figure 5). The surfactant, although helping to form a dye dispersion, might also hinder the diffusion of the dye onto the para-aramid fiber fabrics, even in the presence of soybean oil. The soybean oil pretreatment improved dyeing, which was further improved by longer subsequent NTP treatment times. Evidently, the longer NTP treatment time of 150 s, comparing to the treatment time of 30 s, resulted in higher color strength in the presence or the absence of TWEEN 80 surfactant.

It appeared that the NTP treatment time is crucial to the dyed color strength, especially for the soybean oil pretreated samples. Therefore, Experiment E was designed to optimize the NTP treatment time. In Experiment E, samples were soaked in soybean oil for 15 h, water was used as the dye solvent with a 0.1 wt.% dye concentration and with no other additives and the dyeing temperature was T = 90 °C for 1 h.

**Figure 6:**
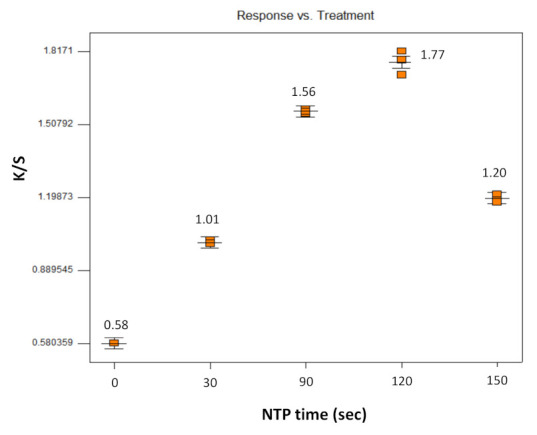
Effect of NTP time on K/S value of soybean oil-soaked para-aramids.

Results of the one-factor factorial design with NTP treatment time varied from 0–150 s are presented in Figure 6. The effects of NTP treatment at these experimental conditions show (Figure 6) that an NTP treatment time of 120 s provided the optimal result in dyeing the soybean oil pretreated samples in terms of color strength as represented by K/S values. NTP treatment times shorter than 120 s achieved dyeing, but to a lesser extent and NTP treatment times longer than 120 s decreased the K/S value, probably due to the degradation of the soybean oil network by the increased exposure to the NTP treatment.

Based on the results of Experiments A–E, Experiment F was designed to optimize dyeing performance by developing a suitable recipe that included auxiliary additives (swelling agent and acetic acid) in the dye bath and a suitable dyeing process that in terms of the dyeing temperature (T = 70 °C or 100 °C) and the effects of a 120 s NTP treatment prior to versus post soaking in soybean oil. The addition of acetic acid into the dye bath brought down the pH to about 3, which should make the conditions favorable for cationic dyes [14]. Results of the 4-factor full factorial design are shown in Table 5.

**Figure 7:**
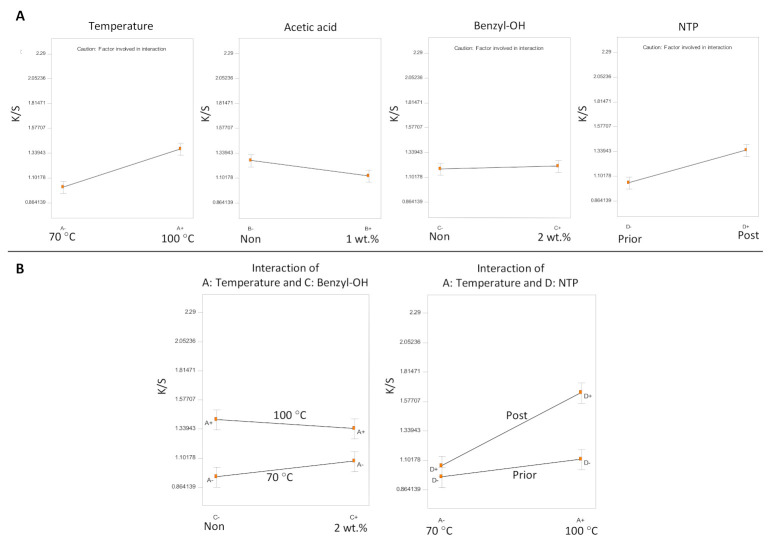
Analysis of Experiment F. (**A**) Main effects of all of the factors and (**B**) the significant interactions.

The half-normal probability plot indicated that impact of the factors is in the order of Dye temperature > NTP > Acetic acid. Even though the overall ANOVA model was significant (*p* < 0.0001), the coefficient for benzyl alcohol was not significant (*t*-test, *p* = 0.63). The only significant interaction term is Temperature × NTP (*t*-test, *p* = 0.0003), while the Temperature × Benzyl–OH (Benzyl alcohol) interaction was marginally significant (*t*-test, *p* = 0.085). Figure 7 plots out the main effects of all the factors along with the two interactions.

Acetic acid was the only factor not involved in any significant interactions (Figure 7A). The addition of 1 wt.% acetic acid in the dye bath slightly decreased the dyeing color strength. All of the other three factors were involved in the two interactions, so their effects are discussed in the context of their interactions (Figure 7B).

The dyeing temperature and NTP after soaking in soybean oil significantly impacted the dyeing results, whereas the use of benzyl alcohol did not significantly influence the dyeing color strength. In Experiment F, the largest K/S value achieved was K/S = 2.28 (sample F15) in dyeing conditions with T = 100 °C in a water-based dye bath, with a pretreatment of soaking in soybean oil followed by a 120 s NTP treatment and no other additives of benzyl alcohol or acetic acid in the dye bath. It is noteworthy that T = 100 °C is the highest temperature that can be used for the water-based dye bath at atmospheric pressure without significant loss of water due to evaporation. In general, dyeing at T = 100 °C was better than dyeing at T = 70 °C. While NTP treatment (120 s) prior to soaking in soybean oil did not show a significant effect on dyeing, NTP treatment after soaking in soybean oil significantly improved the color strength, especially when the dyeing was carried out in the higher temperature regime of T = 100 °C. This clearly points out that the improved dyeing effect should be attributed to the chemical reactions between soybean oil and NTP.

All samples were laundered with a detergent wash for a second time according to the same protocol described in Section 2.5, to determine colorfastness in terms of the change in K/S values (denoted as ΔK/S in Table 5). A negative sign of the ΔK/S value indicates a decrease in K/S value after the second wash with detergent. Generally, higher temperature and NTP treatment after soaking in soybean oil resulted in better dyeing strength and more durable dyeing (colorfastness) after laundering.

**Figure 8:**
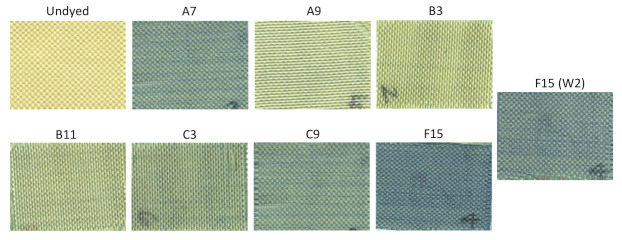
Scanned images comparing dyed samples with the undyed fabric (labels correspond to the sample numbers in Table 1, Table 2, Table 3, Table 4 and Table 5; F15 (W2) denotes sample F15 after second detergent wash).

For the purposes of providing a visual demonstration comparing the dyeing strengths of different treatments, select samples were imaged using a flatbed scanner (Figure 8). Compared with the previous investigation of dyeing para-aramids with this method and using a disperse dye [27], it is demonstrated that the pretreatment of soybean oil followed by NTP treatment method is compatible with dyeing with both disperse dye and cationic dye. Further, this method enables dyeing to a significantly high color strength, although the disperse dye requires glycerol as a dispersant, but water as the solvent is better suited for the use of cationic dye. Taken together, these results demonstrate the potential for this method to replace hazardous chemicals currently used in dyeing practices with renewable materials and environmentally friendly (“green”) technologies, to improve the dyeing of para-aramid textiles.

### 3.2. FTIR Analysis

**Figure 9:**
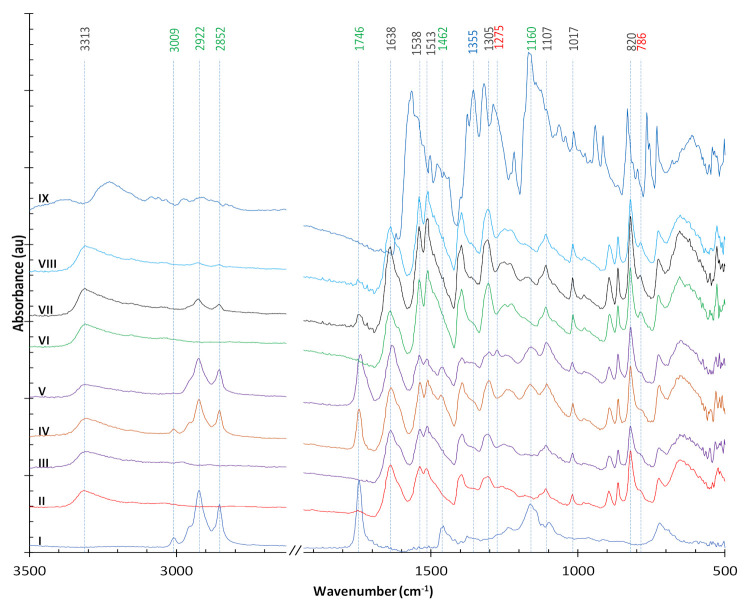
FTIR spectra of the para-aramid fabrics, materials and after certain treatments as follows: (**I**) Soybean oil; (**II**) Para-aramid fabric (untreated); (**III**) Para-aramid fabric treated with NTP; (**IV**) Para-aramid fabric after soaking in soybean oil; (**V**) Para-aramid fabric after soaking in soybean oil followed by a subsequent NTP treatment; (**VI**) Para-aramid fabric dyed with NTP as the only pretreatment; (**VII**) Para-aramid fabric dyed after soaking in soybean oil (without an NTP treatment); (**VIII**) Para-aramid fabric dyed after soaking in soybean oil and an NTP treatment; and (**IX**) Victoria Blue R cationic dye.

As shown in Figure 9, the following characteristic peaks of soybean oil (line I) are well represented: 3009 cm^−1^ (=C–H stretching of aliphatic alkenes) in unsaturated fatty acids, 2922 cm^−1^ (–CH_2_ asymmetric stretching), 2852 cm^−1^ (–CH_3_ symmetric stretching), 1746 cm^−1^ (–C=O triglycerides carbonyl stretching), 1462 cm^−1^ (–CH_2_ antisymmetric deformation) and 1160 cm^−1^ (C–O stretching in the esters) [30,31,32,33].

The spectrum of untreated para-aramids (line II) has main peaks at 3313 cm^−1^ (–N–H stretching), 1638 cm^−1^ (amide I C=O stretching) and 1538 cm^−1^ (–N–H deformation) [34]. The absorption bands at 1513 cm^−1^ (amide II), at 1017 cm^−1^ and at 820 cm^−1^ deriving from the C–H bonds on the para-aromatic rings and the absorption band at 1305 cm^−1^ deriving from the C–N bond stretch of amide III of the para-aramid fiber fabrics can be considered as internal standards for para-aramid [35]. NTP treatment of the para-aramid sample (line III) did not induce discernible changes in the observed FTIR peaks. A similar result was observed in Kašparová et al. [36], which also reported some changes in meta-aramids after NTP treatment and may reflect the different internal molecular structures between meta-aramids and para-aramids that make para-aramids more difficult to dye even with an NTP treatment.

The sample in line IV for the para-aramid fabric soaked in soybean oil shows peaks characteristic of soybean oil and of the para-aramids. Subsequent NTP treatment induces some important changes in the observed spectrum (line V). First, the unsaturated fatty acids peak at 3009 cm^−1^ decreases, but the other major characteristic peaks of soybean oil were still visible. This observation suggests that the double bonds in the unsaturated fatty acids were rapidly consumed by the NTP treatment, presumably by reaction with the plasma’s reactive oxygen and nitrogen species (RONS). Second, there were changes in the 1380–1250 cm^−1^ region, including the formation of a small peak appearing at 1275 cm^−1^, which is assigned to the C–C stretching vibration of the C–C(=O)–C groups in aliphatic ketone molecule [33]. These results suggest that the plasma’s RONS attack the C=C double bonds, concomitant to the decrease in the peak at 3009 cm^−1^ (=C–H stretching of aliphatic alkenes). However, the ketone molecule is unstable. A likely explanation is that high-energy reactive oxygen species in the NTP (generated with ambient air as the feedgas) attack the electron rich double bonds in the unsaturated fatty acids and form epoxides, although the characteristic oxirane absorption peak typically observed at ~822 cm^−1^ would be obscured by the strong para-aramid absorption peak at 820 cm^−1^. The –C=O triglycerides carbonyl stretching and C–O stretching in the esters at 1746 cm^−1^ and 1160 cm^−1^, respectively, remain strong in line V, indicating that the NTP did not break the ester bonds in soybean oil.

Strong acids such as phosphoric acid and sulfuric acid have been used to pretreat para-aramids for the improvement of dyeing or interfacial bonding; a new absorption peak at ~3440 cm^−1^ (which is ascribed to the hydroxyl group O–H) or the shift and broadening of the 3313 cm^−1^ (–N–H stretching) band was reported, along with some decrease in mechanical strength. This distinct change was explained as a consequence of the increased number of –OH groups in the modified para-aramids due to hydrolysis [2,3]. However, this effect is not observed for the para-aramid sample pretreated with acetic acid in this study, and the acetic acid pretreatment did not significantly improve the color strength of dyeing.

FTIR spectra for dyed para-aramid fabric samples (Figure 9) are shown in line VI (with only NTP pretreatment), line VII (with soybean oil soaking but no NTP) and line VIII (with soybean oil soaking and NTP). The three spectra all have a new peak emerging at 786 cm^−1^, which is typically the region of C–H bending for 1,3-disubstituted or 1,2,3-trisubstituted aromatic rings, possibly indicating a new bond forming on the para-aromatic rings. Otherwise, the spectrum of the sample dyed only with NTP pretreatment (line VI) is not much different from that of undyed para-aramids (line II or III). The para-aramid fabric sample in line VII (pretreated with soybean oil soaking and without subsequent NTP treatment, showed peaks characteristic of soybean oil that were significantly diminished. NTP treatment after oil soaking induced further decreases in the signal intensity of soybean oil (line VIII). In addition to reactions with NTP, there are two possible explanations for the diminished oil signal: (1) unfixed oil could be washed off after dyeing and subsequent washing with detergent; and (2) the triacylglycerol could be hydrolyzed at the dyeing conditions resulting in the destruction of ester bonds, as indicated by the diminished peaks at 1746 cm^−1^ and 1160 cm^−1^.

The FTIR spectrum of the Victoria Blue R is also shown in Figure 9 (line IX). The para-aramid fabric samples showed a low uptake of dye, and most of the dye peaks were obscured by the characteristic peaks of the para-aramids and the oil. Accordingly, no significant dye peaks were detected in the dyed samples, except for a small shoulder at 1355 cm^−1^ in the spectra of the dyed samples (lines VII and VIII).

It is likely that the soybean oil well diffused throughout the tightly woven fabric and adsorbed onto the surfaces of yarns and fibers, and the NTP treatment induced the formation of a polymerized network in situ, enabling dyeing to a higher color strength. The unsaturated fatty acids in soybean oil play important roles in the dyeing process. Cross-linking/polymerization by the action of reactive plasma species are possible, because hydrogenation, nitration and epoxidation reactions have been observed along with polymerization under atmospheric NTP [37]. In a study on the tribological properties of air plasma polymerized soybean oil, Zhao et al. [33] concluded that the free radicals in the long-chain oil molecules, which were formed by the opening of the double bonds under the plasma conditions, could capture the reactive O and N species to produce and incorporate carbonyl, organic amine and nitrogen heterocyclic groups into the polymerized oil network. Moreover, depending on the duration of the NTP treatment in the dyeing procedure, the soybean oil might undergo processes such as complex oxidations, decomposition or fragmentation, to form reactive intermediate sites (e.g., ethers, furans, peroxides, carboxylic acids or 1,2,4-trioxolane [38,39]) on the para-aramid fiber fabrics that serve as catalysts or ligands to bind dye molecules.

## 4. Conclusions

Using a cationic dye and an environmentally friendly alternative dyeing method, we found that the best formulation for dyeing para-aramid textiles involved (i) pre-treating the para-aramid fiber fabrics with soybean oil followed by a brief NTP treatment, and (ii) using water at T = 100 °C as the dye solvent and omitting auxiliary chemical additives. Dyeing temperature and NTP treatment time were the most important factors, with the dyeing temperature of T = 100 °C resulting in higher color strength (larger K/S values), and the optimized NTP treatment time of 120 s for soybean oil pretreated para-aramids. These conditions achieved a K/S value up to 2.28, which is significantly larger than the K/S ~1 for untreated samples. For comparison, NTP alone or the use of the auxiliary additives of acetic acid and benzyl alcohol only slightly improved dyeing with the cationic dye. As a dye solvent, glycerol was inferior to water for dyeing with the cationic dye, and the addition of the surfactant TWEEN 80 in the dye bath negatively impacted the dyeing performance.

In the present study, FTIR analysis revealed that NTP induced chemical reactions in soybean oil on the surface of para-aramid fabrics were responsible for significantly improving the color strength. These chemical reactions likely involved high-energy, short-lived reactive plasma species that first attacked the double bonds in the unsaturated fatty acids. While the results of this study provide ample evidence that the pretreatment of soaking in soybean oil and ensuing NTP treatment improved dyeing color strength, determining the chemical pathways that led to better dyeing warrant further research, to control these processes with other dyestuffs. Future research along this line of sustainable dyeing method will aim at deriving more functionalities of soybean oil on the surface of para-aramids to further improve the dyeing strength and colorfastness.

## Figures and Tables

**Table 1:** Experiment A: experimental design and results ^@^.

Sample	Dye Solvent	Pretreatment	NTP Time (s)	Swelling Agent	K/S	K/S Std *
A1	Water	None	60	None	1.26	0.009
A2	Water	None	0	None	1.20	0.054
A3	Water	Acetic acid	60	None	1.22	0.011
A4	Water	Acetic acid	0	None	1.12	0.018
A5	Water	None	60	Benzyl–OH	1.44	0.003
A6	Water	None	0	Benzyl–OH	1.56	0.026
A7	Water	Acetic acid	60	Benzyl–OH	1.74	0.021
A8	Water	Acetic acid	0	Benzyl–OH	1.59	0.016
A9	Glycerol	None	60	None	0.42	0.005
A10	Glycerol	None	0	None	0.39	0.009
A11	Glycerol	Acetic acid	60	None	0.45	0.004
A12	Glycerol	Acetic acid	0	None	0.40	0.005
A13	Glycerol	None	60	Benzyl–OH	0.41	0.002
A14	Glycerol	None	0	Benzyl–OH	0.42	0.003
A15	Glycerol	Acetic acid	60	Benzyl–OH	0.48	0.019
A16	Glycerol	Acetic acid	0	Benzyl–OH	0.47	0.004

^@^ Experiment conducted at soaking time in 20 wt.% acetic acid solution = 15 h, dye concentration = 0.1 wt.%, Benzyl alcohol concentration = 2 wt.%, T = 140 °C, dyeing time = 1 h. * Values represent mean and standard error of 3 replicates.

**Table 2:** Experiment B: experimental design and results ^@^.

Sample	Dye Solvent	Pretreatment	Swelling Agent	NTP Time (s)	K/S	K/S Std *
B1	80% Glycerol	Soy oil	Benzyl–OH	60	0.70	0.015
B2	80% Glycerol	Soy oil	Benzyl–OH	0	0.57	0.012
B3	80% Glycerol	Soy oil	None	60	0.79	0.014
B4	80% Glycerol	Soy oil	None	0	0.56	0.004
B5	80% Glycerol	None	Benzyl–OH	60	0.55	0.003
B6	80% Glycerol	None	Benzyl–OH	0	0.53	0.004
B7	80% Glycerol	None	None	60	0.46	0.017
B8	80% Glycerol	None	None	0	0.44	0.013
B9	50% Glycerol	Soy oil	Benzyl–OH	60	0.84	0.005
B10	50% Glycerol	Soy oil	Benzyl–OH	0	0.67	0.009
B11	50% Glycerol	Soy oil	None	60	0.94	0.001
B12	50% Glycerol	Soy oil	None	0	0.92	0.005
B13	50% Glycerol	None	Benzyl–OH	60	0.81	0.014
B14	50% Glycerol	None	Benzyl–OH	0	0.70	0.010
B15	50% Glycerol	None	None	60	0.72	0.006
B16	50% Glycerol	None	None	0	0.82	0.013

^@^ Experiment carried out with soaking in soybean oil time = 15 h, dye concentration = 0.1 wt.%, Benzyl alcohol concentration = 2 wt.%, T = 140 °C and dyeing time = 1 h. * Mean and standard error of 3 replicates.

**Table 3:** Experiment C: experimental design and the results ^@^.

Sample	Dye Solvent	Pretreatment	Swelling Agent	NTP Time (s)	K/S	K/S Std *
C1	50% Glycerol	Soy oil	Benzyl–OH	90	0.91	0.021
C2	50% Glycerol	Soy oil	Benzyl–OH	0	0.60	0.002
C3	50% Glycerol	Soy oil	None	90	1.06	0.045
C4	50% Glycerol	Soy oil	None	0	0.57	0.006
C5	50% Glycerol	None	Benzyl–OH	90	0.96	0.009
C6	50% Glycerol	None	Benzyl–OH	0	0.74	0.015
C7	50% Glycerol	None	None	90	1.04	0.027
C8	50% Glycerol	None	None	0	0.66	0.013
C9	Water	Soy oil	Benzyl–OH	90	1.66	0.262
C10	Water	Soy oil	Benzyl–OH	0	1.15	0.008
C11	Water	Soy oil	None	90	1.72	0.072
C12	Water	Soy oil	None	0	0.86	0.008
C13	Water	None	Benzyl–OH	90	1.68	0.024
C14	Water	None	Benzyl–OH	0	1.31	0.104
C15	Water	None	None	90	1.09	0.034
C16	Water	None	None	0	0.92	0.012

^@^ Experiment was carried out with soaking in soybean oil for time = 15 h, dye concentration = 0.1 wt.%, Benzyl alcohol concentration = 2 wt.%, T = 90 °C and dyeing time = 1 h. * Mean and standard error of 3 replicates.

**Table 4:** Experiment D: experimental design and the results ^@^.

Sample	Surfactant	Pretreatment	NTP Time (s)	K/S	K/S Std *
D1	None	None	30	0.81	0.006
D2	None	Soy oil	30	1.01	0.008
D3	None	None	150	0.97	0.008
D4	None	Soy oil	150	1.20	0.017
D5	TWEEN	None	30	0.61	0.010
D6	TWEEN	Soy oil	30	0.60	0.002
D7	TWEEN	None	150	0.79	0.003
D8	TWEEN	Soy oil	150	0.84	0.005

^@^ Experiment carried out with soaking in soybean oil for time = 15 h, dye solvent = water, dye concentration = 0.1 wt.%, T = 90 °C and dyeing time = 1 h. * Mean and standard error of 3 replicates.

**Table 5:** Experiment F: experimental design and the results ^@^.

Sample	Temperature	Acetic Acid	Swelling Agent	NTP	K/S	K/S Std *	ΔK/S ^#^
F1	70 °C	Yes	Benzyl–OH	Post	1.08	0.016	−0.08
F2	70 °C	Yes	Benzyl–OH	Prior	0.91	0.022	−0.13
F3	70 °C	Yes	None	Post	0.99	0.005	−0.17
F4	70 °C	Yes	None	Prior	0.91	0.045	−0.27
F5	70 °C	No	Benzyl–OH	Post	1.18	0.008	−0.15
F6	70 °C	No	Benzyl–OH	Prior	1.12	0.025	−0.27
F7	70 °C	No	None	Post	0.96	0.023	−0.06
F8	70 °C	No	None	Prior	0.93	0.007	−0.31
F9	100 °C	Yes	Benzyl–OH	Post	1.55	0.012	−0.04
F10	100 °C	Yes	Benzyl–OH	Prior	1.15	0.016	−0.20
F11	100 °C	Yes	None	Post	1.30	0.007	−0.10
F12	100 °C	Yes	None	Prior	1.06	0.020	−0.17
F13	100 °C	No	Benzyl–OH	Post	1.45	0.010	−0.12
F14	100 °C	No	Benzyl–OH	Prior	1.21	0.068	−0.16
F15	100 °C	No	None	Post	2.28	0.064	−0.09
F16	100 °C	No	None	Prior	1.01	0.013	−0.17

^@^ Experiment carried out with soaking in soybean oil for time = 15 h, dye solvent = water, dye concentration = 0.1 wt.%, dyeing time = 1 h, Additive concentrations = 1 wt.% Acetic acid and/or 2 wt.% Benzyl alcohol and the NTP treatment time = 120 s prior to or post soaking in soybean oil for 15 h. * Mean and standard error of 3 replicates. ^#^ The change in K/S value was calculated by subtracting the K/S after the 1st detergent wash from the K/S value measured after the 2nd detergent wash.

## Data Availability

The data presented in this study are available on request from the corresponding author.
